# Mapping the landscape of chromatin dynamics during naïve CD4+ T-cell activation

**DOI:** 10.1038/s41598-021-93509-w

**Published:** 2021-07-08

**Authors:** Muhammad Munir Iqbal, Michael Serralha, Parwinder Kaur, David Martino

**Affiliations:** 1 Telethon Kids Institute, Northern Entrance, Perth Children’s Hospital, 15 Hospital Avenue, Nedlands, Perth, WA 6009 Australia; 2 UWA School of Agriculture and Environment, The University of Western Australia, 35 Stirling Highway, Nedlands, Perth, WA 6009 Australia; 3 Centre for Food and Allergy Research, Murdoch Children’s Research Institute, University of Melbourne, Flemington Road, Parkville, VIC 3053 Australia

**Keywords:** Lymphocyte activation, Epigenetics in immune cells

## Abstract

T-cell activation induces context-specific gene expression programs that promote energy generation and biosynthesis, progression through the cell cycle and ultimately cell differentiation. The aim of this study was to apply the omni ATAC-seq method to characterize the landscape of chromatin changes induced by T-cell activation in mature naïve CD4+ T-cells. Using a well-established ex vivo protocol of canonical T-cell receptor signaling, we generated genome-wide chromatin maps of naïve T-cells from pediatric donors in quiescent or recently activated states. We identified thousands of individual chromatin accessibility peaks that are associated with T-cell activation, the majority of which were annotated intronic and intergenic enhancer regions. A core set of 3268 gene promoters underwent chromatin remodeling and concomitant changes in gene expression in response to activation, and were enriched in multiple pathways controlling cell cycle regulation, metabolism, inflammatory response genes and cell survival. Leukemia inhibitory factor (LIF) was among those factors that gained the highest accessibility and expression, in addition to IL2-STAT5 dependent chromatin remodeling in the T-cell activation response. Using publicly available data we found the chromatin response was far more dynamic at 24-h compared with 72-h post-activation. In total 546 associations were reproduced at both time-points with similar strength of evidence and directionality of effect. At the pathways level, the IL2-STAT5, KRAS signalling and UV response pathways were replicable at both time-points, although differentially modulated from 24 to 72 h post-activation.

## Introduction

Naïve CD4+ T-cells circulate through the periphery in an actively maintained state of quiescence, ready to mount a robust immune response to pathogens. Quiescent T-cells maintain a tightly condensed chromatin architecture^1^ and cellular program of low energy expenditure whilst surveying for cognate antigen^2^, and rapidly undergo substantial re-programming following activation, transitioning toward highly proliferative effector cells. Activation of naïve T-cells initiates rapid functional adaptations which, over the course of days, evolves into heterogenous effector fates with unique helper and regulatory functions with the potential for establishing long-lived memory phenotypes. Activated T-cells rapidly increase nutrient uptake, ramp up translational activity and switch to glycolytic pathways to provide the energy required to support cell growth^3^, a massive proliferative response and the acquisition of effector functions. These adaptive changes are well understood to be underpinned by epigenetic^4^, metabolic^3^, transcriptional and proteomic^2^ changes.

At the nuclear level, T-cell receptor (TCR) signaling induces dynamic re-positioning of nucleosomes at promoters and enhancers to allow for transcriptional changes^5^. These dynamic changes in the chromatin landscape enable interactions between sequence-specific transcription factors (TF) with regulatory DNA elements. Although promoters are the primary sites of transcription initiation, enhancers are major determinants of cell-specific transcriptional and physiological adaptations^6^. The assay for transposase-accessible chromatin (ATAC-seq) has gained in popularity as a method to map chromatin accessibility corresponding to TF binding sites and nucleosome positioning^7^ at the genome-wide scale, due to its high resolution and low cell input, enabling ex vivo analyses^8,9^. A more recent variant of the ATAC-seq method known as omni-ATAC has demonstrated advantages for removing unwanted mitochondrial reads and exhibits better performance on fixed and flash-frozen material^10^. ATAC-seq integrated with TF binding motifs has proven increasingly useful for uncovering the dynamic changes in enhancer landscapes and predicting key regulatory events that bring about chromatin remodeling. The dynamic remodeling of enhancer landscapes and differential TF motif usage is a characteristic of distinct T-helper subsets^11^. The majority of data available to date has been performed on neonates^12^, adults^2,13^ or murine cells^1,14–16^, and there is a paucity of data on infants and young children. Thus, our goal was to examine the utility of omni-ATAC for characterizing chromatin dynamics in paediatric bio-banked samples. We have previously described deficiencies in T-cell activation transcriptional networks and activation-induced regulation of DNA methylation in young infants who developed IgE-mediated food allergy^17,18^. Studying T-cell activation responses at the molecular level has translational potential for understanding disease mechanisms and uncovering novel molecular targets. Thus, the main aim of this pilot study was to assess the utility of omni-ATAC for uncovering *cis-*regulatory elements in cryopreserved paediatric samples.

In this study we isolated mature naïve T-cells from six healthy paediatric donors and studied chromatin dynamics in the canonical T-cell receptor signaling pathway using an identical protocol as published previously by us^19^. This allowed us to analyze stimulation-dependent chromatin changes in the context of previously collected transcriptomic data. Our data are largely consistent with previous studies^20^, demonstrating the utility of omni-ATAC for characterizing the enhancer landscape in paediatric bio-banked samples, as a prelude to future studies of disease mechanism.

## Results

### Post-alignment QC

**Figure 1:**
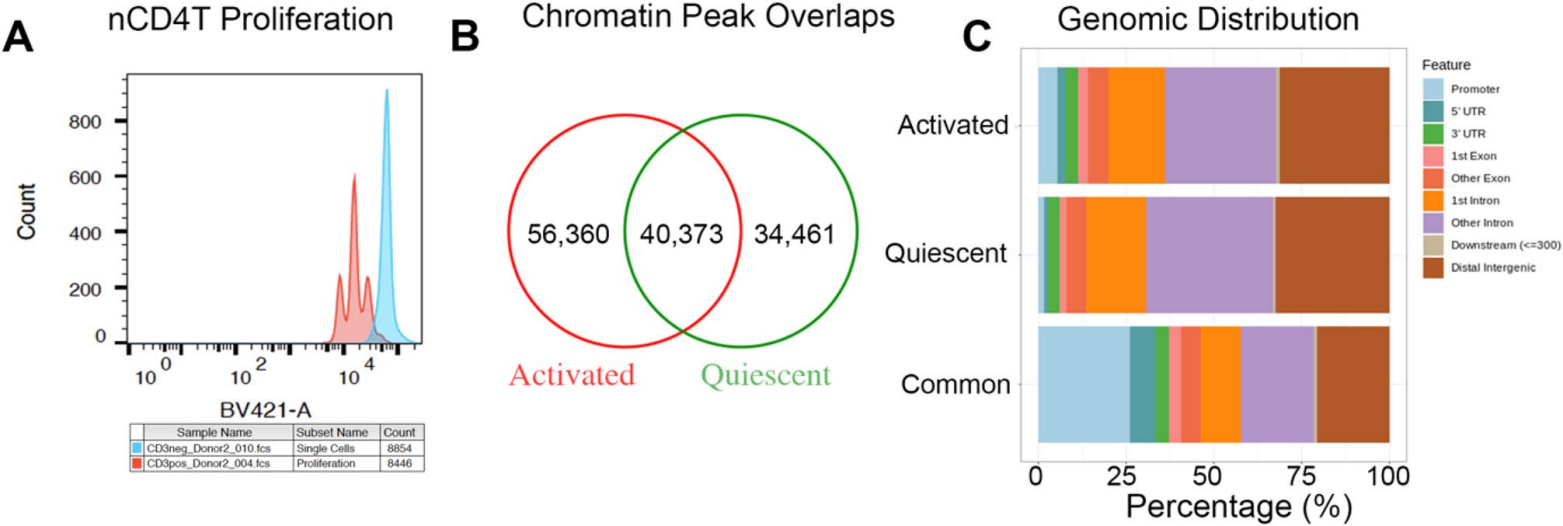
Activation of nCD4T induces widespread changes in chromatin accessibility. **(A)** nCD4T stained with CellTrace Violet and stimulated in culture for 3 days. Discreet peaks represent successive generations of live cells. The unstimulated parent generation is show in blue. **(B)** Venn diagram showing counts of chromatin accessibility peaks. **(C)** Annotation of consensus chromatin accessibility peaks to genomic regions of the hg38 genome.

We isolated mature naïve T-cells from six healthy infants and studied chromatin dynamics in the canonical T-cell receptor signaling pathway using an identical protocol as published previously by us^19^. Naïve T-cells were activated with anti-CD3/anti-CD28 beads for 72 h (activated nCD4T) with matched un-stimulated control condition (quiescent nCD4T). Using cell tracing dyes this protocol results in 3–4 distinct T-cell divisions expanding the clonal population on average by twofold (expansion index 2.044 [range 1.80–2.23], Fig. 1A). After 72 h all cells were recovered for chromatin profiling. Previous analysis demonstrated activated cells harvested in this phase represent a transitional population of highly proliferative early effector phenotypes^18^. We generated maps of genome-wide chromatin accessibility to identify epigenomic elements that bring about the stimulation response in resting and activated cells. Post-alignment quality control indicated high mapping efficiency with overall alignment rates 96% or higher. Fragment length distribution plots yielded high resolution of nucleosome-free and nucleosome-occupied reads. Mitochondrial DNA was detected in 23–39% of reads in our data set (30–40% of duplicates) despite the omni-ATAC protocol. Reads were highly enriched at universal DNAse1 hypersensitivity regions identified by the ENCODE consortium^21^ and enhancer regions indicative of regulatory DNA elements. Activated nCD4T cells exhibited a higher number of reads at promoter regions compared with resting nCD4T (Fig. S1).

### Open chromatin peak occupancy analysis

Compared to resting nCD4T, the genomes of activated cells were more accessible as evidenced by a larger number of detected open chromatin peaks. We identified 74,834 consensus peaks in quiescent nCD4T and 96,733 peaks in activated nCD4T, with 40,373 common peaks (Fig. 1B). We annotated consensus peaks to the hg38 reference genome and examined the distribution of peaks across genomic features. Peaks commonly identified in both activated and resting cells were more likely to be observed in promoter regions compared with peaks unique to each condition (Fig. 1C). Peaks unique to activation were more frequently annotated to promoter regions compared with peaks unique to quiescent cells (6.2% activated; 2.2% quiescent), the latter were more enriched at intronic regions (31.6% activated; 36% quiescent) (Fig. 1C). The overall distribution of peaks was consistent with the typical pattern of ATAC peaks representing a mixture of different *cis-*regulatory elements such as enhancers and promotors^22^.

### Differential binding analysis

**Figure 2:**
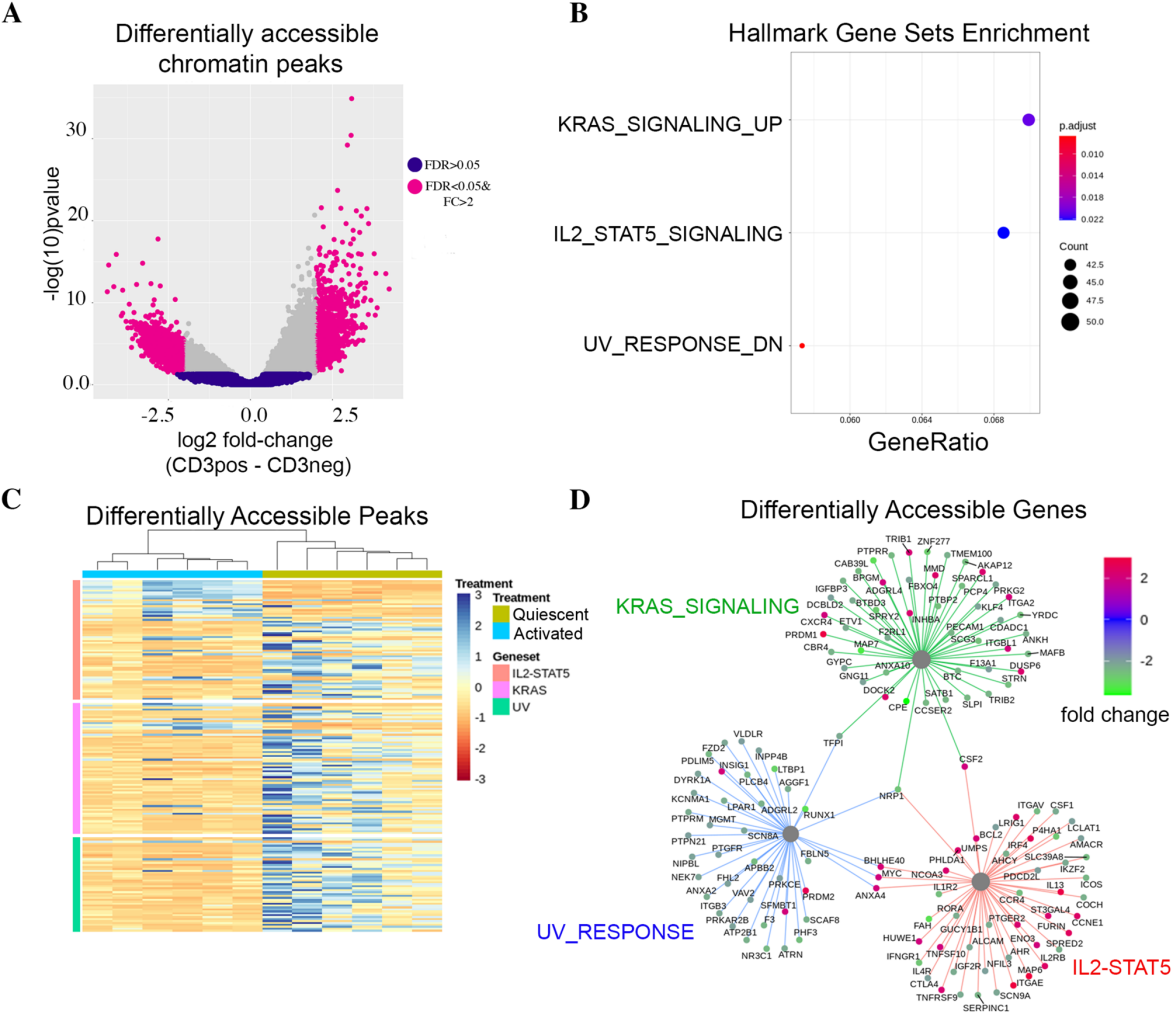
Differential accessibility of chromatin peaks in activated versus resting cells.** (A)** Volcano plot of differentially accessible peaks. Each data point represents a consensus peak. **(B)** Gene sets enrichment analysis of differentially accessible peaks. The dot plot shows significantly enriched pathways (hallmark collection) at the FDR < 0.05 level. **(C)** Cluster heatmap of 130 differentially accessible peaks annotated to enriched pathways. Rows represent peaks, columns represent samples. Cells are colored according to row-scaled read counts. Columns are clustered by Euclidean distance with complete linkage. **(D)** Gene concept network plots the links between differentially accessible genes (edges) and their associated enriched pathways (nodes). Edges are colored according to fold-change values.

We quantified the number of differentially accessible activation-induced chromatin changes by formally testing MACS2 peaks between quiescent and activated nCD4T. Short-term activation of the T-cell receptor induced substantial changes in the chromatin landscape comprising 43,269 chromatin peaks (FDR < 5%) that were differentially accessible, of which 5,607 exhibited a minimum absolute log2 fold change in accessibility of ± 2.0 (Fig. 2A, Table S1). Of the 5,607 differentially accessible peaks, a total of 1,089 peaks gained accessibility in activated nCD4T whilst 4,518 peaks reduced in accessibility. We observed that only 30% of peaks called unique to one condition (in Fig. 1B) are identifiable as significantly differentially accessible, which is partly due to the stringency of the differential testing, and that differentially accessible sites are more likely to be called in peaks unique to treatment groups (Fig S2). Hypergeometric testing revealed significant (FDR < 0.05) enrichment of peaks within three core signal transduction pathways: IL2-STAT5, KRAS signaling and UV response genes (Fig. 2B, Table S4). Peaks within these pathways (n = 130) were extracted from the total data set and cluster heatmap analysis was used to examine sample clustering and fold changes. As expected, activated and quiescent cells formed distinct clusters and scaled fold-change values revealed the KRAS and UV response pathways generally decreased in accessibility, whilst the IL2 pathway was dynamically modulated exhibiting both increases and decreases in accessibility (Fig. 2C,D).

### Relationship to transcriptional changes

**Figure 3:**
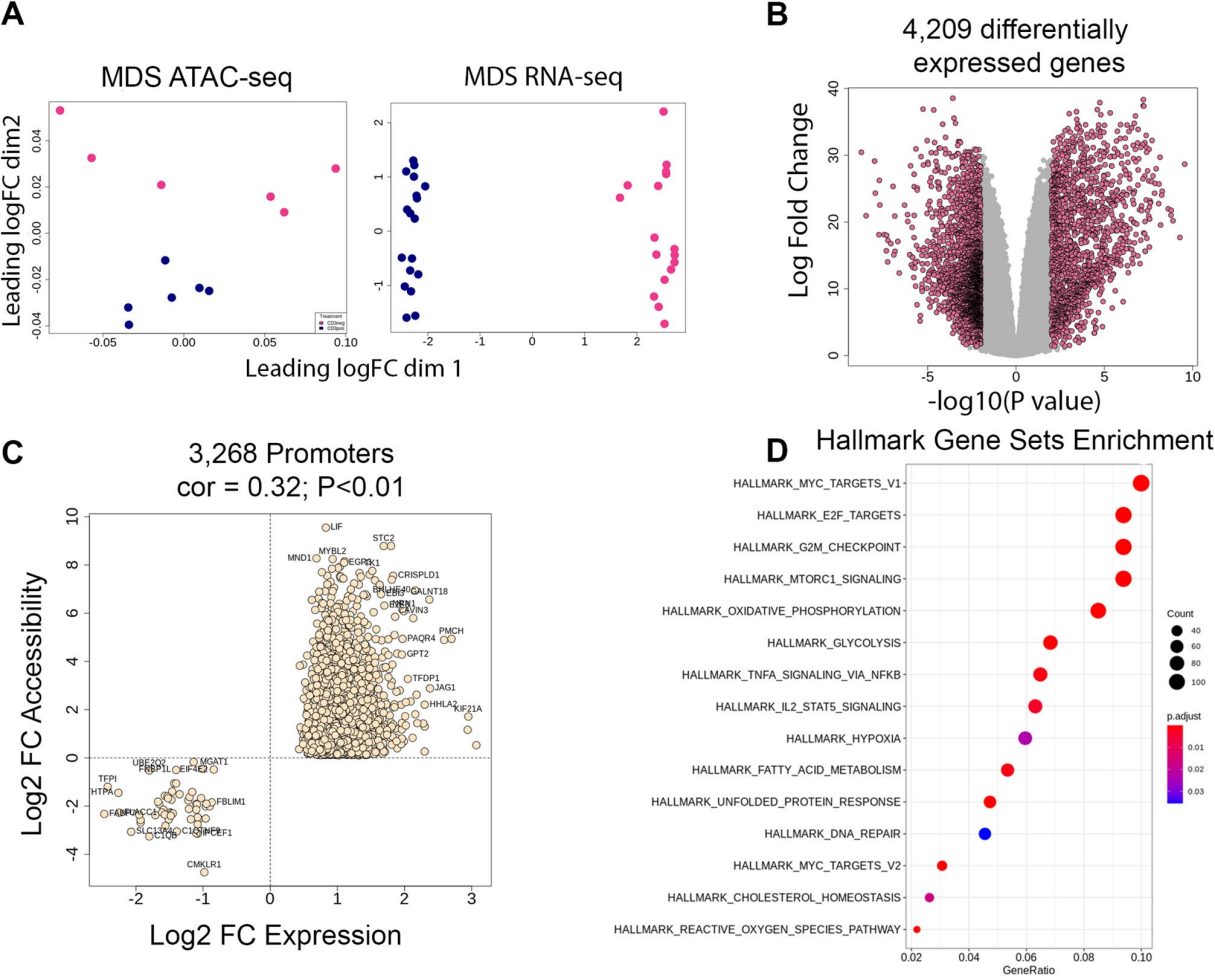
Analysis of gene expression at stimulus-dependent accessible regions. **(A)** Multidimensional scaling analysis of accessibility (all detected peaks) and expressed genes (all unique transcripts). **(B)** Volcanoplot of differentially expressed genes (FDR < 0.05 & log2FC + /− 2; pink dots). **(C)** Scatterplot of accessibility log fold changes and gene expression fold changes (act—quiescent) for FDR-significant hits annotated to promoter regions. *Cor* Pearson’s correlation coefficient. **(D)** Dotplot of enriched Hallmark gene sets among 3268 promoter regions.

We next sought to determine how differentially accessible regions associated with activation are related to changes in gene expression. For this analysis we used transcriptomic data from our previous naïve CD4T study with harmonized laboratory stimulation protocol (GSE114064). Multidimensional scaling analysis demonstrates that T-cell activation results in distinct chromatin conformations with concomitantly unique transcriptional signatures (Fig. 3A). In total 4209 genes were differentially expressed in activated versus quiescent T-cells (FDR < 0.05 & log fold-change + /− 2, Fig. 3B). We merged the 5607 differentially accessible peaks with the RNA-seq data set and examined the relationship between log fold changes in accessibility and log fold changes in gene expression. For peaks annotated to promoter regions (219 peaks) significant positive correlation with gene expression was observed (Pearson’s correlation = 0.32, P < 0.01, Fig. S3). This correlative relationship did not hold for peaks outside of promoter regions. To more broadly identify gene promoters that exhibit coordinate pattern of regulation, we merged the summary statistics for ATAC-seq and RNA-seq data sets based on shared gene identifier and calculated z-scores for the log fold change value for each gene and peak combination. The combination of gene and peak has a positive z-score if gene expression and accessibility change in the same direction between treatment conditions. In total 3,268 gene promoters exhibited coordinate regulation of accessibility and expression (FDR < 0.05 ATAC & RNA-seq) (Fig. 3C, Table S2). Enrichment analysis of coordinately regulated genes identified multiple pathways including those related to cell cycle regulation (MYC, E2F, G2M checkpoint), cell metabolism (MTORC1, oxidative phosphorylation, glycolysis, fatty acid synthesis, hypoxia, reactive oxygen, cholesterol homeostasis), inflammatory response (TNFa signaling, IL2-STAT) and cell survival (unfolded protein response, DNA repair) (Fig. 3D, Table S4).

### Reproducibility with other data sets

**Figure 4:**
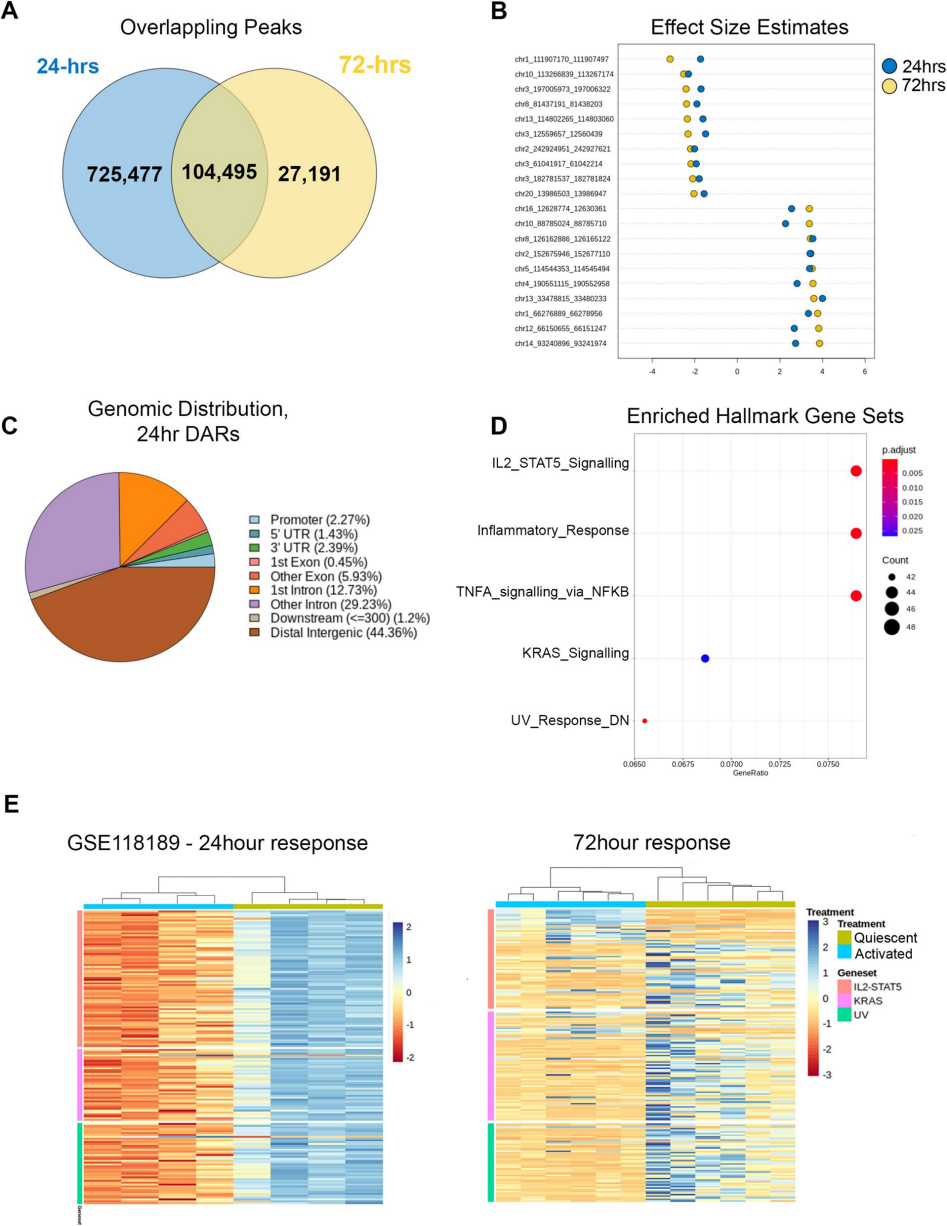
Reproducibility analysis with GSE118189. **(A)** Venn diagram of total detected overlapping peaks. **(B)** Dot chart of logFC effect size estimated for top 20 reproducible peaks. Data for all peaks are shown in Fig S4. **(C)** Pie chart of genomic distribution of 4,387 differentially accessible regions (DARs) at 24-h post activation (GSE118189). **(D)** Gene sets enrichment analysis of 4387 DARs. **(E)** Cluster heatmap of differentially accessible peaks annotated at 24 and 72 h. Rows represent peaks, columns represent samples. Cells are colored according to row-scaled read counts. Columns are clustered by Euclidean distance with complete linkage.

To determine the reproducibility of our observations we analyzed ATAC counts from a similar study of resting and activated nCD4T from four donors from a 24-h stimulation (as opposed to 72-h) using the same antigen (GSE118189^20^). The activation response at 24-h was far more dynamic comprising of 829,942 accessibility peaks compared to 131,686 peaks at 72-h. In total 104,495 peaks in the 72-h data set overlapped with the 24-h data set, which was more than expected by chance (hypergeometric P < 0.01, Fig. 4A). We performed a lookup analysis of our 5,607 differential peaks at 72 h post activation by converting the genomic coordinates to the hg19 genome and identifying overlapping regions in the replication data set. In total 3,126 peaks were available for differential testing in the 24-h data set. Of these, 546 associations were replicated at FDR < 0.05 and had a directionally similar effect at 24- and 72-h post-activation (Fig. 4B, Fig. S4). We next tested whether similar pathways were enriched in the 24-h data by analyzing peaks overlapping with the 72-h data set (104,495 peaks). Differentially testing over these peaks identified 4,387 associations with T-cell activation (FDR ≤ 0.05 & logFC + /− 2). These peak coordinates were converted to the hg38 genome and the genomic distribution was broadly similar to that observed in activation specific peaks at 72-h (Fig. 4C). Hypergeometric testing of peak overlaps with Hallmark biological pathways identified the same core enriched pathways (IL2-STAT5, KRAS, UV response) as were initially identified as differentially accessible at 72-h, as well as additional pathways involved in inflammatory response and TNF signaling via NFκB (Fig. 4D, Table S4). We extracted the peaks within the IL2-STAT5, KRAS and UV pathways (n = 157 peaks) and performed cluster heatmap analysis to determine whether the pathways were changing in the same direction. This visualization analysis revealed that at 24-h, these pathways were highly up-regulated, but by 72 h they were dynamically changing being downregulated or a combination of both (Fig. 4E). Taken together, we were able to draw similar biological conclusions and directly replicated more hits than would be expected by chance in GSE118189 indicating features of our data set were replicable in under different experimental setting.

## Discussion

T-cell activation induces global remodeling of chromatin accessibility in an orderly and timely manner. These epigenetic changes are coincidental with specific gene regulatory networks that bring about changes in cellular metabolism, proliferative capacity and effector function^11^. In this study we compared genome-wide chromatin accessibility maps between quiescent and activated naïve CD4+ T-cells. Consistent with previous studies^1^ we found that TCR signaling induces wide-spread de-condensation of chromatin, as evidence by substantially higher (~ 22,000) open chromatin peaks detected in activated cells. We found that stimulus-dependent chromatin accessibility changes were enriched at enhancer and intronic regions to a large extent, and less so for gene promoters, indicative of a network of *cis-*regulatory elements that bring about the activation response. In our data set, at 72-h post activation we found 3 major pathways underwent dynamic chromatin remodeling including IL-2-STAT5, KRAS signaling and UV response. These biological pathways were replicated in GSE118189, a slightly different data set which is a snapshot naïve T-cell activation response at 24 h. The replicability of these pathways at both time points indicated their major role in T-cell activation, although we found differences in the 24-h response. Specifically, many genes within these pathways were up-regulated in the short-term, then dynamically modulated by 72 h indicating a change in the response. Using previously collected transcriptomic data, we identified a core set of ~ 3,000 promoters that undergo significant chromatin remodeling and changes in gene expression. In this data set, targets of the IL2-STAT5 and TNFα-NFκB signaling pathways were highly enriched among differentially accessible and expressed genes. These are extremely well characterized transcriptional pathways in T-lymphocyte responses^23^. STAT5 is a well-described early remodeling factor invoked by IL-2 family cytokines which establishes enhancer landscapes within T-cell lineages^24^. It has a known role in recruiting chromatin remodelers in Treg cells^25^ and promotes accessibility at the IL-9 locus in Th17 cells^26^. NFκB induction occurs in response to TCR signaling following intracellular calcium mobilization and is needed for the induction of inflammatory responses^27^. Nuclear translocation of NFκB binds to κB elements in target inflammatory response genes, turning them on^28^. Remodeling of these pathways at the chromatin level represents a key regulatory mechanism of the inflammatory response^29^. In addition to these inflammatory pathways, we identified chromatin remodeling at multiple pathways that mediated T-cell metabolic states and control of the cell cycle.. Collectively, these data provide a ‘birds eye’ view of the major pathways subject to chromatin level regulation in response to TCR activation. We found these biological observations were replicable in the slightly different data set GSE118189, with a subset of our hits directly reproduced in this data set.

## Materials and methods

### Subject selection

Subjects were recruited through Princess Margaret Hospital in Perth, Western Australia as part of a community-based program of allergy prevention. All subjects used in this study underwent prospective clinical assessments at 1, 2.5 and 5 years of age, including phenotyping for allergic outcomes and general health and donated venous blood for cryopreservation according to institutional ethics committees. Inclusion criteria for selecting biospecimens for this study included equal numbers of males (n = 3) and females (n = 3), subjects were 1-year of age at time of biospecimen collection, subjects did not receive any interventions, subjects had more than 1 vial of cryopreserved peripheral blood mononuclear cells (PBMC) in the biobank. Exclusion criteria included any congenital malformations, any primary immune deficiency or clinically significant illness that would affect normal hematopoietic development. General characteristics of the cohort are provided in Table S3. All methods and protocols were approved by Princess Margaret Hospital Human Research Ethics Committee and informed consent was obtained from the legal guardian of all participants. All experiments were performed in accordance with relevant guidelines and regulations.

### Isolation, activation and expansion of naïve CD4+ T-cells

Cryopreserved PBMC were thawed in RPMI media (Gibco) supplemented with 10% fetal bovine serum (FBS), Pen-Strep and benzonase (25 U/mL) maintained in a 37-degree water bath. After thawing, cells were washed twice, counted and viability checked by trypan blue. Cell recoveries ranged from 8 to 20 million PBMC with viabilities higher than 90%. Naïve CD4+ T-cells (CD3 + CD4+ CD45RA + CD45RO−) were purified from PBMC using the EasySep Human CD4+ T-cell Isolation Kit (Stemcell Technologies) to > 95% purity according to manufacturer’s instructions. Yield of naïve T-cells ranged from 1 to 2.5 million cells. Naïve CD4+ T cells were pre-labelled with 5 mM CellTrace Violet division tracking dye (Thermo Fisher) according to manufacturer’s instructions and seeded into 96-well polystyrene plates at 80,000 cells per well in RPMI media with 10% FBS, Pen-Strep and human recombinant interleukin-2 (210 U/mL, R&D systems). For activation, 2 µL of Human T-cell Activator Dynabeads CD3/CD28 (Life Tech) was added to each well reserved for activation, with an equal number of un-activated wells. Cells were incubated for 72 h at 37 and 5% CO_2_ before harvesting. At culture end-point, cells were thoroughly resuspended, and beads were removed with replicate wells combined into a single tube for ATAC-seq. A proportion of replicate wells was reserved for proliferation analysis on the BD Fortessa cytometer with 405 nm excitation and 450/40 bandpass emission filter.

### Omni ATAC-seq

We employed the omni-ATAC method of Corces. 80,000 viable naïve T-cells were pelleted and lysed in lysis buffer containing 10 mM Tris–HCl, 10 mM NACl, 3 mM MgCL_2_, 0.1% NP40, 0.1% Tween20 and 0.01% Digitonin for 3 min on ice. Cells were washed with 1 mL of cold wash buffer (lysis buffer without NP40 or Digitonin) and nuclei were pelleted in a centrifuge at 800 RCF for 10 min at 4 degrees. Pelleted nuclei were transposed with Tn5 transposase (Illumina) in TD buffer (Illumina) supplemented with Digitonin (0.1%) and Tween20 (0.01%) for 30 min at 37. Transposed DNA was purified using Zymo DNA Clean and Concentrator-5 Kit (Zymo research) according to manufacturer’s instruction. DNA recoveries were measured on the Qubit fluorometer (Invitrogen). Library amplification was performed using Nextera DNA library prep kit with Nextera Index Kit (Illumina) as per manufacturers instruction. The number of PCR amplification cycles was determined by qRT-PCR using Quanitfast SYBR Green PCR mastermix (Qiagen) and Nextera Primer I5 and I7 Indexes for 5 cycles. The number of additional cycles was determined by a second round of qPCR performed on partially amplified libraries based on the CT value reading taken at 1/3 the fluorescence curve. Two step size selection was performed using AMPure XP beads (Beckman Coulter). Libraries were run on the LabChip GXII fragment analyser and quantitated on the Qubit fluorometer. Libraries were shipped on ice to Novogene (China) for pooling and sequencing on 2 lanes of the Illumina HiSeq at 2 × 150 paired end reads to generate 50 million reads per sample.

### Bioinformatics

Raw fastq files were analysed using the Multiqc program to generate QC metrics and were processed using the ENCODE official ATAC-seq pipeline version 1.4 specified here. Briefly, adapters detection and trimming were performed using cutadapt (1.91.) and trimmed reads were aligned to hg38 genome using the Bowtie2 (2.2.6) aligner. Mapping statistics were generated with SAMtools (1.7) and SAMstats (0.2.1). Post-alignment filtering of duplicates was performed using Picard (1.126) and bedtools (2.26). Aligned reads were shifted + 4 bp for the forward strand and − 5 bp for the reverse strand. Fragment length statistics were generated using Picard (1.126). Peak calling was conducted using MACSv2 (2.1.0) and blacklisted regions were filtered using bedtools (2.26). Irreproducibility analysis was performed on pseudoreplicates using phantompeakqualtools (1.2.1) and IDR (2.0.4) on 300 K MACS2 peaks using a threshold of 0.05. Reads were annotated to ENCODE regions using python scripts and bedtools (2.26).

### Data analysis

All data analyses were conducted in R version 4.0.2^30^. MAC2 peaks were coerced to a peakset object using Diffbind (2.16)^31^. Consensus peaksets were derived for activated and quiescent cells defined by peak presence in more than half the replicates in each group. Peaks were annotated to the hg38 genome using ChIPseeker (1.24)^32^. Enrichment analysis of peaks in Hallmark genesets^33^ as well as Gene Sets Enrichment Analysis was conducted using the clusterProfiler package (3.16)^34^ using a threshold of FDR < 0.05 to define enriched pathways. Normalized read counts for consensus peaks were computed for each sample using Diffbind, and differential accessibility between activated and quiescent T-cells was determined using a matched pairs t-test using the edgeR package (3.30)^35^. Peaks were declared differentially accessible at the genome-wide level of false discovery rate adjusted P-value < 0.05 and those exhibiting a log twofold change of ± 2 or greater were further analysed. Peak signal tracks were generated using the rtracklayer package (1.48)^36^. RNAseq data from GSE114064 were downloaded for a subset of age-matched healthy control infants and TMM normalized count data were voom transformed using limma (3.44.3)^37^. Differentially expressed genes were declared by comparing transcript expression levels between activated and quiescent T-cells using a matched-pairs t-test (limma) at the genome-wide level of FDR-adjusted P-value < 0.05 and log2 fold change + /− 2 or greater^30^. Merging of ATAC-seq peaks and RNA-seq transcripts was performed using the mergeByOverlaps function in the GenomicRanges R package (1.4)^38^ using any overlap between genomic intervals. Raw counts from data set GSE118189 were analyzed for ‘Naïve_Teff’ condition only. Counts were transformed to log-2 counts per million with variance stabilization as implemented in the limma package using the voom function. To determine the significance of overlapping peaks between data sets, hypergeometric testing of overlaps was performed assuming a total test size of 2.9 million possible accessibility sites as reported in Thurman et al.^22^. To identify co-regulated gene sets after merging RNA-seq and ATAC-seq data sets, we computed Z-scores from the log fold change combinations of each peak *p* and gene *g* defined as:

$${Z}_{p,g}\,\,=\,\,\frac{{logFC}_{g}^{(RNA)}}{sd\left({logFC}_{g}^{RNA}\right)}\bullet \frac{{logFC}_{g,p}^{(Peak)}}{sd\left({logFC}_{g,p}^{(Peak)}\right)}$$

## Supplementary Information

Supplementary Figure S1.

Supplementary Figure S2.

Supplementary Figure S3.

Supplementary Figure S4.

Supplementary Legends.

Supplementary Table S1.

Supplementary Table S2.

Supplementary Table S3.

Supplementary Table S4.

Supplementary Table S5.

## Data Availability

The data sets generated for this study are deposited in the Gene Expression Omnibus Repository under accession number GSE157174.
