# Supplementary figures and images for: Mapping the landscape of chromatin dynamics during naïve CD4+ T-cell activation
Source: Sci Rep. 2021 Jul 8;11:14101. doi: 10.1038/s41598-021-93509-w (PMC8266878; doi:10.1038/s41598-021-93509-w)

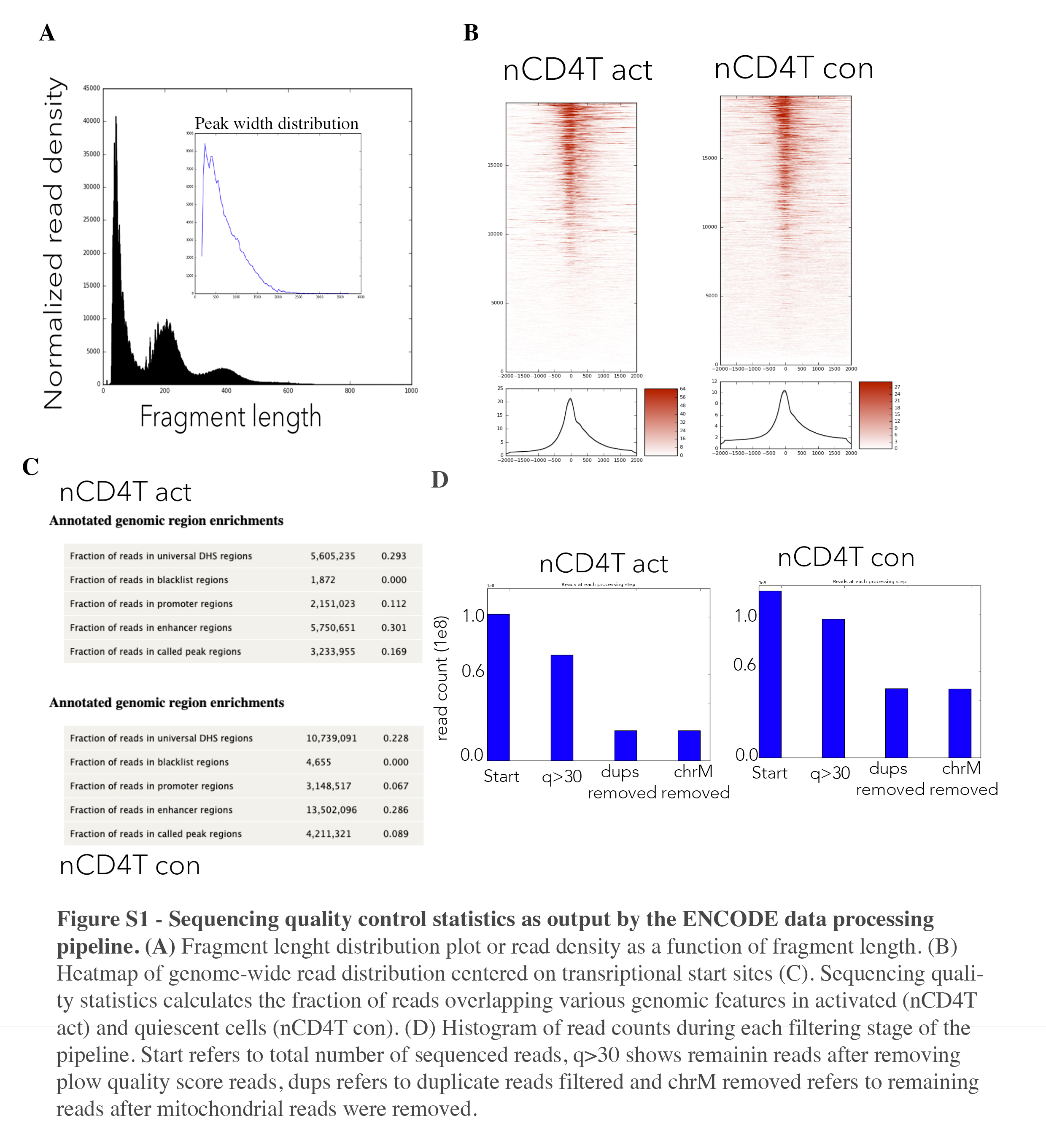

Supplement: Supplementary file 1 — Supplementary Figure S1. [file 41598_2021_93509_MOESM1_ESM.jpg]

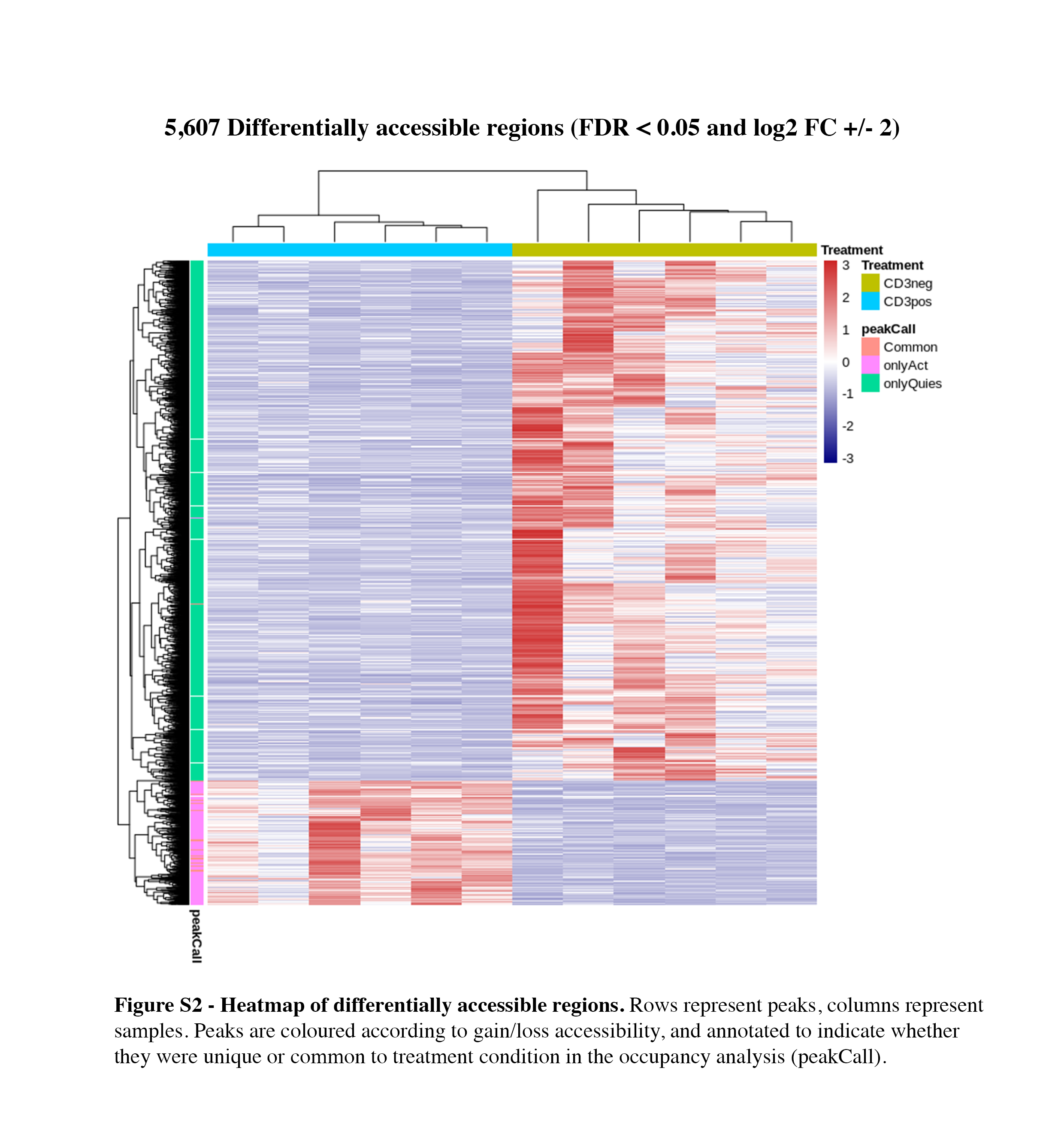

Supplement: Supplementary file 2 — Supplementary Figure S2. [file 41598_2021_93509_MOESM2_ESM.tif]

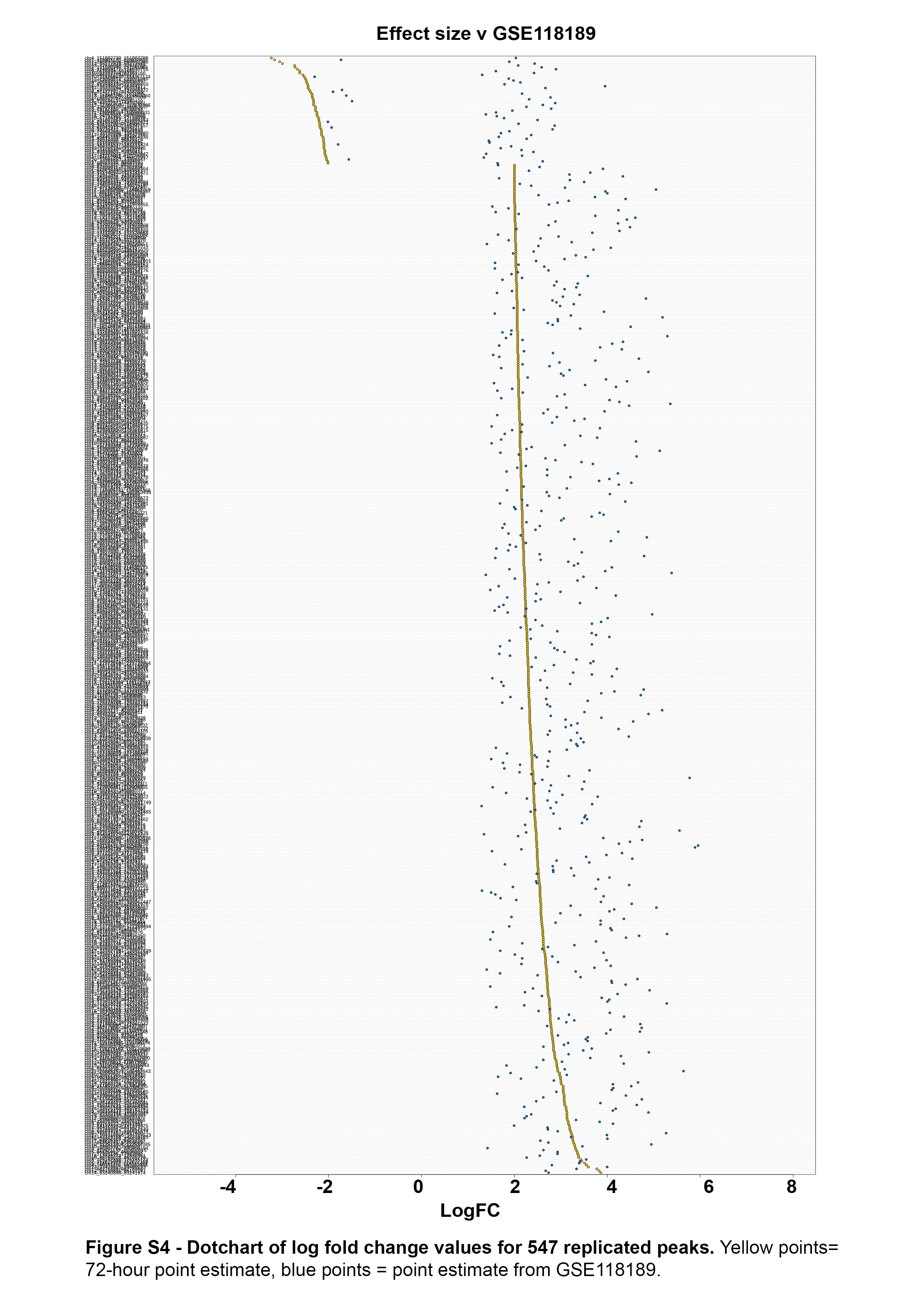

Supplement: Supplementary file 4 — Supplementary Figure S4. [file 41598_2021_93509_MOESM4_ESM.tif]
