# Supplementary material for: Mapping the landscape of chromatin dynamics during naïve CD4+ T-cell activation
Source: Sci Rep. 2021 Jul 8;11:14101. doi: 10.1038/s41598-021-93509-w (PMC8266878; doi:10.1038/s41598-021-93509-w)
Supplement: Supplementary file 3 — Supplementary Figure S3. [file 41598_2021_93509_MOESM3_ESM.pdf]

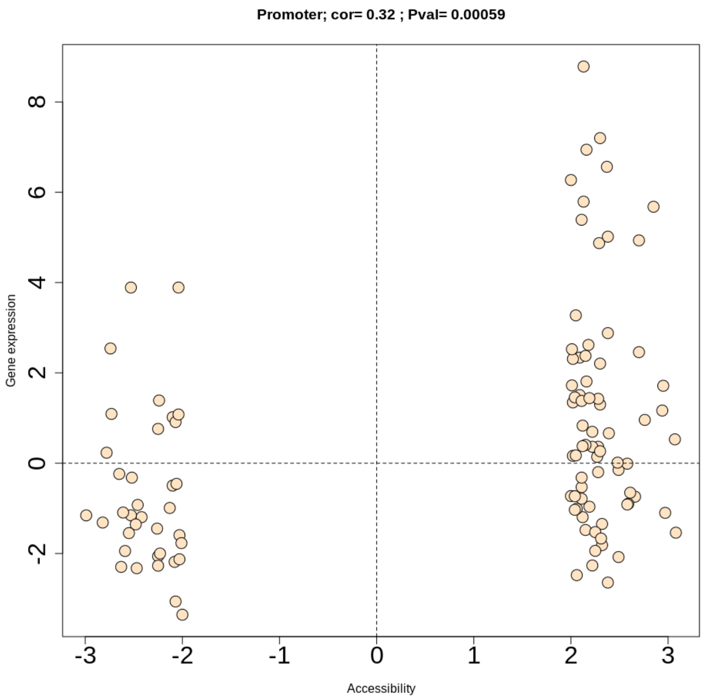

Figure S3 – Scatterplot of differentially accessible promoters and gene expression. Plot show the relationship between log fold-change values for 219 gene promoters identified as differentially accessible in response to T-cell activation. Cor = Pearson's correlation.
