# Supplementary material for: Mapping the landscape of chromatin dynamics during naïve CD4+ T-cell activation
Source: Sci Rep. 2021 Jul 8;11:14101. doi: 10.1038/s41598-021-93509-w (PMC8266878; doi:10.1038/s41598-021-93509-w)
Supplement: Supplementary file 5 — Supplementary Legends. [file 41598_2021_93509_MOESM5_ESM.docx]

SUPPLEMENTARY INFORMATION

Mapping the landscape of chromatin dynamics during naïve CD4+ T-cell activation.

**Authors:** Muhammad Munir Iqbal^1^, Michael Serralha^1^, Parwinder Kaur ^2^, David Martino^1,2,3,*^.

**Affiliations:**

^1^Telethon Kids Institute, Northern Entrance, Perth Children's Hospital, 15 Hospital Avenue, Nedlands, Western Australia, 6009, Australia

^2^Faculty of Science, UWA School of Agriculture and Environment, The University of Western Australia, 35 Stirling Highway, Nedlands, Western Australia, 6009, Australia

^3^ Centre for Food and Allergy Research, Murdoch Children’s Research Institute, University of Melbourne, Flemington Road, Parkville, Victoria, 3053, Australia

*To whom correspondence should be addressed: [David.Martino@telethonkids.org.au](mailto:David.Martino@telethonkids.org.au), Telethon Kids Institute, Perth Children’s Hospital, 15 Hospital Avenue, Nedlands, Perth, WA, 6009.

Running title: Chromatic Dynamics of T-cell Activation

Key Words; T-cell, chromatin

Table S1- 5,607 differentially accessible peaks

Table S2 – 3,268 co-regulated promoters associated with nCD4T activation

Table S3 – General characteristics of the population studied

Table S4 – Gene sets enrichment analysis statistics

Figure S1 – Sequencing QC statistics as output by the ENCODE data processing pipeline.

Figure S2 – Heatmap of differentially accessible regions

Figure S3 – Scatterplot of differentially accessible promoters and gene expression.

Figure S4 – Dotchart of log fold change values for all replicated peaks.
