# Supplementary material for: Mapping the landscape of chromatin dynamics during naïve CD4+ T-cell activation
Source: Sci Rep. 2021 Jul 8;11:14101. doi: 10.1038/s41598-021-93509-w (PMC8266878; doi:10.1038/s41598-021-93509-w)
Supplement: Supplementary file 8 — Supplementary Table S3. [file 41598_2021_93509_MOESM8_ESM.docx]

| Age (m) | 12 |
| --- | --- |
| Sex (% female) | 50 |
| Caesarean birth (%) | 17 |
| Term birth (%) | 100 |
| Family history allergy (%) | 17 |
| Allergic sensitization | 50 |

**Table S3 – General characteristics of population studied**
